# Eye contact, a fundamental building block of social behavior, engages single unit activity in the monkey amygdala

**DOI:** 10.1186/1471-2202-13-S1-P131

**Published:** 2012-07-16

**Authors:** Clayton P Mosher, Prisca E Zimmerman, Katalin M Gothard

**Affiliations:** 1 Graduate Interdisciplinary Program in Neuroscience, The University of Arizona, Tucson, AZ 85724, USA; 2 Department of Physiology, The University of Arizona, Tucson, AZ 85724, USA

## 

**Figure 1:**
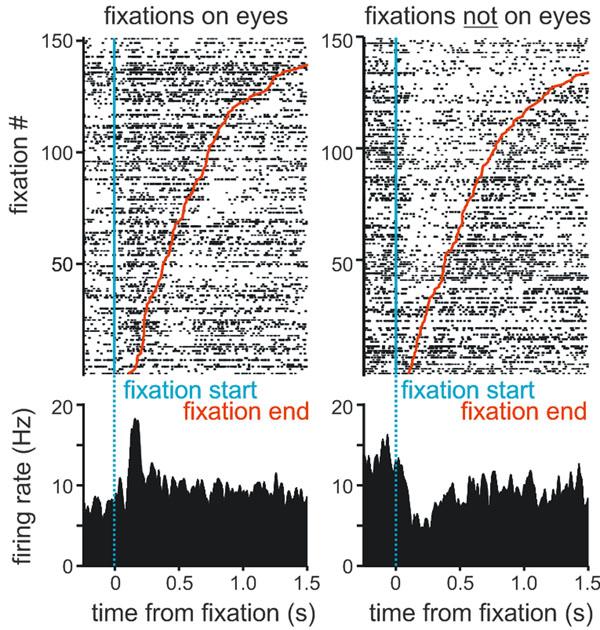
Rasterplot and peristimulus time histogram of a single unit recorded from the monkey amygdala. This neuron had an increased firing rate that was maintained for the entire duration that the viewer monkey fixated on the eyes of the movie monkey (left panel). This same neuron was inhibited when the viewer looked anywhere else (right panel).

In primates, the meaningful use of facial signals and eye contact is a prerequisite for normal social behavior. Just by looking at the face of another monkey, an individual can determine its age, sex, dominance status, health, etc. Facial expressions are useful to determine the emotional state and possible intentions of others. Eye contact facilitates affiliative behaviors such as facial mimicry in the context of mother-infant interactions, but also conveys threats and dominance status in adult-adult interactions. The neural circuitry that underlies these behaviors is largely unknown. One goal of our research is to determine the role of the primate amygdala in basic aspects of social communication that involve looking at the eyes of other individuals. To achieve this goal we developed an experimental paradigm that elicits reliably and reproducibly several aspects of social behavior including eye contact, facial mimicry, and gaze following [1]. Additionally, we recordeded single unit activity from the monkey amygdala to determine which of these social components (if any) reliably induce neural responses. Species-specific and socially meaningful behaviors were elicited using naturalistic videos that depicted unknown monkeys displaying neutral, agonistic, or affiliative behaviors. Each video contained segments of displays when the movie monkey's eye gaze was directed toward the viewer monkey. The eye movements of the viewer monkey were co-registered with each movie frame and with multiple channels of single unit activity recorded from the amygdala. We found that 23/123 (19%) of neurons in the amygdala discharged selectively or exclusively when the viewer monkey looked at the eyes of the movie monkey (Figure 1). These neurons had a response latency of 100-150ms from the start of fixation on the movie monkey’s eyes. They exhibited either excitatory (13/23, 57%) or inhibitory responses to looking at the eyes, and either no response (or a polar opposite response) to looking at other parts of the face or body. Higher responses occurred when the movie monkey's eye gaze was directed at the viewer (eye contact) than for averted gaze. A subset of neurons showed phasic responses indicating that eye contact had been established, whereas others showed tonic, sustained changes in firing for the entire duration of the fixation. We conclude that the amygdala is likely an important center for the elaboration of a fundamental building block of social behavior: looking at the eyes of others and establishing eye contact.
